# Editorial: Revisiting the limits of plant life - plant adaptations to extreme terrestrial environments relating to astrobiology and space biology

**DOI:** 10.3389/fpls.2023.1267183

**Published:** 2023-08-17

**Authors:** Agata K. Zupanska, Carmen Arena, Gustavo E. Zuñiga, Angelica Casanova-Katny, Johanna D. Turnbull, León A. Bravo, Patricio Ramos, Hang Sun, Vladimir V. Shishov

**Affiliations:** ^1^Carl Sagan Center, SETI Institute, Mountain View, CA, United States; ^2^Department of Biology, University of Naples Federico II, Naples, Italy; ^3^Laboratorio de Fisiología y Biotecnología Vegetal, Universidad de Santiago de Chile, Santiago, Chile; ^4^Department of Environmental Sciences, Faculty of Natural Resources, Catholic University of Temuco, Temuco, Chile; ^5^School of Earth, Atmospheric and Life Sciences (SEALS), University of Wollongong, Wollongong, NSW, Australia; ^6^University of La Frontera, Temuco, Chile; ^7^Instituto de Ciencias Biológicas, Universidad de Talca, Talca, Chile; ^8^Vicerrectoría de Investigación y Postgrado, Universidad Católica del Maule, Talca, Chile; ^9^Kunming Institute of Botany, Chinese Academy of Sciences (CAS), Kunming, China; ^10^School of Fundamental Biology and Biotechnology, Siberian Federal University, Krasnoyarsk, Russia

**Keywords:** plant, extreme environment, astrobiology, deep space exploration, limits of life

Plants were essential to the early evolution of terrestrial life and colonization of the young Earth (Kapoor et al., 2023). Plant communities continue to colonize and transform our planet including the newest ecosystems formed post-glaciation, restoring those degraded by human activities and adapting to changing ecological conditions (Huston and Smith, 1987; Chapin et al., 1994; Yuan et al., 2020; Heim et al., 2021). Plants cannot move away from a harmful stimulus, and thus, have evolved remarkable strategies to survive and eventually thrive in harsh environments. Today, humanity is on the verge of exploring our solar system and beyond, eager to discover, answer fundamental questions, and search for extraterrestrial forms of life. Undoubtedly, plants are key organisms to successful deep space missions and independence from the provision of terrestrial resources, whether for long duration interplanetary travel or establishing permanent settlements. We can employ terrestrial bio-design principles to mimic plant colonization on Earth and explore adaptations to novel ecosystems. This can be combined with precise molecular tools to enable long-term human space exploration. With this thought in mind, we have collated articles focusing on terrestrial plants from extreme environments and their adaptations to harsh conditions. This collective knowledge will advance the selection of desired plant characteristics relevant to human space missions and assist in the identification of valuable adaptive mechanisms that can be genetically engineered in targeted space food plants. Furthermore, terrestrial plants from the edge of habitability define limits for terrestrial forms of life and thus can direct efforts for the search for life beyond Earth (McKay, 2014; Huwe et al., 2019).

In this Research Topic, De Micco et al. present an exhaustive review on the hazards of cosmic ionizing radiation to human exploration and discuss potential roles for plants in mitigating this Research Topic. The authors highlight difficulties in studying ionizing radiation in deep space that stem from deploying inadequate radiation sources, limited access to ion beam accelerators, and lack of uniformity in radiation doses and dose rates (acute, chronic) across studies. The authors expand on the remarkable resistance of plants growing in regions of high radioactivity and the possibility of using such plants in shielding or for pharmaceutical countermeasures. Next, Molina-Montenegro et al. demonstrate the benefits of co-culturing relevant crops with endophytic fungi in simulated deep space environments to augment plant performance in high UV radiation, low temperature, and low water availability. In this instance, plants from mesic habitats were combined with fungi from the extreme environment of the Atacama Desert, which as the driest place in the world and also experiences excessive solar irradiance. Increased survival in crops inoculated with desert fungi was related to higher biomass, antioxidant content and nutritional quality in deep space-like environmental conditions compared to non-inoculated plants. Thus, Molina- Montenegro et al. contribute to developing the use of symbionts from extreme terrestrial environments to improve the efficiency of crop cultivation in space.

In another study, Gong et al. challenged emerging seedlings with growth mediums of varying particle size, porosity, and compactness, which is pivotal to understanding the intricacies of germinating seeds in extraterrestrial regolith in future space farms. Extraterrestrial regolith on anhydrous and airless celestial bodies is formed by vastly different processes than terrestrial soils. These processes include large and small meteoroid impacts, star plasma winds, and cosmic ionizing radiation, and cause distinct mechanical, physical, and hydraulic properties. Gravitational effects further impact regolith formation by affecting collision and aggregation velocities, which influences the values of bulk densities and the porosity of the outermost regolith layers. Using CT scans corroborated by mathematical models, Gong et al. demonstrate that underground seedlings search for a path of least resistance irrespective of the distance to the surface, and that high porosity and low compactness of the growth medium are conducive to seedling emergence. This finding informs plant cultivation on the Moon using *in situ* regolith. Due to micrometeoroid impacts, lunar regolith has very fine, low- density particles with complex shapes, sharp jagged edges, and a high angular surface. According to Gong et al., these tight and abrasive pore spaces could impede seedling path to the surface. However, the lower gravitational field on the Moon results in much lower regolith bulk densities, hence low compactness, which, according to the findings of Gong et al., is likely to promote seedling emergence.

Another study in this Research Topic addressed the quest for extraterrestrial life. Battistuzzi et al. explore if the light spectrum emissions from faint and cool stars is sufficient for oxygenic photosynthesis in cyanobacteria. This is relevant to the search for oxygen biosignatures on exoplanets orbiting M-dwarf or Red Dwarf stars in habitable zones. These stars emit light at the far-red (700–750nm) and infrared (750–1000nm) wavelengths, unlike the Sun, which emits light predominantly in the visible and UV spectrum. On Earth, photosynthesizing plants harvest solar blue and red light for the light reactions of photosynthesis, so the capacity for low energy photons at longer wavelengths to photolyze H_2_O was unknown. Battistuzzi et al. show that cyanobacteria can acclimate to the simulated M-dwarf light spectrum by elevating the concentration of pigments capable of quenching low-energy photons and by increasing the efficiency of utilizing the sparse high-energy photons. There was no difference in biomass production nor O_2_ release between cyanobacteria grown under either light spectrum. This demonstrates that the photosynthetic apparatus, which evolved in the earliest terrestrial life forms, could perform in the presence of cool and dim starlight. As such, Battistuzzi et al. contribute a rare physiological analysis to theoretical models evaluating the feasibility of M-dwarf stars to support photosynthesis. This informs and legitimizes the search for oxygen biosignatures.

This Research Topic was conceived with the intent to present research, methods, and reviews to further our understanding of plant survival in harsh or novel environments, and that can be extrapolated to the conditions found in deep space. As humanity leaves Earth’s protective influence to explore the solar system and beyond, we require the knowledge and ideas developed by plant scientists, such as those assembled in this Research Topic, to take plants with us and deploy them to perform mission-critical functions. Once at the destination, guided by the knowledge of plant evolutionary solutions, we will know where to look for extraterrestrial life.

## Author contributions

AZ: Writing – original draft. CA: Writing – review & editing, Data curation. GZ: Writing – review & editing. AC-K: Writing – review & editing. JT: Writing – review & editing. LB: Writing – review & editing. PR: Writing – review & editing. HS: Writing – review & editing. VS: Writing – review & editing.
